# Diaqua­tetra­kis­(1,3-di-4-pyridylpropane-κ*N*)­manganese(II) bis­(perchlorate) ses­qui­hydrate

**DOI:** 10.1107/S1600536811041511

**Published:** 2011-10-22

**Authors:** Hai-Wei Kuai, Xiao-Chun Cheng

**Affiliations:** a Faculty of Life Science and Chemical Engineering, Huaiyin Institute of Technology, Huaian 223003, People’s Republic of China

## Abstract

In the title complex, [Mn(C_13_H_14_N_2_)_4_(H_2_O)_2_](ClO_4_)_2_·1.5H_2_O, the Mn^II^ ion is coordinated by four N atoms from four different 1,3-di-4-pyridyl­propane mol­ecules and two O atoms from two coordinated water mol­ecules, leading to a distorted MnN_4_O_2_ octa­hedral geometry. Each 1,3-di-4-pyridyl­propane ligand displays a monodentate coordinating mode. In the crystal, there exist O—H⋯O, O—H⋯N and C—H⋯O hydrogen bonds. The perchlorate anions and the coordinated and lattice water mol­ecules play an important role in the formation of these hydrogen bonds. One of the two lattice water molecules shows half-occupancy.

## Related literature

For a related structure, see: Zheng *et al.* (2007 ▶).
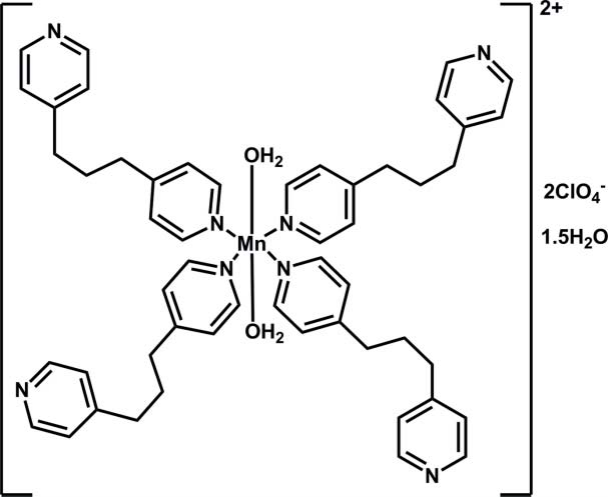


## Experimental

### 

#### Crystal data

[Mn(C_13_H_14_N_2_)_4_(H_2_O)_2_](ClO_4_)_2_·1.5H_2_O
*M* *_r_* = 1108.94
Orthorhombic, 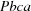

*a* = 19.0755 (10) Å
*b* = 15.9409 (9) Å
*c* = 37.533 (2) Å
*V* = 11412.9 (11) Å^3^
*Z* = 8
Mo *K*α radiation
μ = 0.39 mm^−1^
*T* = 293 K
0.30 × 0.30 × 0.10 mm

#### Data collection

Bruker APEXII CCD area-detector diffractometer
Absorption correction: multi-scan (*SADABS*; Sheldrick, 1996 ▶) *T*_min_ = 0.892, *T*_max_ = 0.962
61756 measured reflections
10065 independent reflections
6106 reflections with *I* > 2.σ(*I*)
*R*_int_ = 0.113

#### Refinement

*R*[*F* ^2^ > 2σ(*F* ^2^)] = 0.057
*wR*(*F* ^2^) = 0.188
*S* = 1.01
10065 reflections
676 parameters
H-atom parameters constrained
Δρ_max_ = 0.66 e Å^−3^
Δρ_min_ = −0.47 e Å^−3^

### 

Data collection: *APEX2* (Bruker, 2008 ▶); cell refinement: *SAINT* (Bruker, 2008 ▶); data reduction: *SAINT*; program(s) used to solve structure: *SHELXS97* (Sheldrick, 2008 ▶); program(s) used to refine structure: *SHELXL97* (Sheldrick, 2008 ▶); molecular graphics: *DIAMOND* (Brandenburg, 2000 ▶); software used to prepare material for publication: *SHELXTL* (Sheldrick, 2008 ▶).

## Supplementary Material

Crystal structure: contains datablock(s) I, global. DOI: 10.1107/S1600536811041511/pv2456sup1.cif

Supplementary material file. DOI: 10.1107/S1600536811041511/pv2456Isup2.cdx

Structure factors: contains datablock(s) I. DOI: 10.1107/S1600536811041511/pv2456Isup3.hkl

Additional supplementary materials:  crystallographic information; 3D view; checkCIF report

## Figures and Tables

**Table 1:** Hydrogen-bond geometry (Å, °)

*D*—H⋯*A*	*D*—H	H⋯*A*	*D*⋯*A*	*D*—H⋯*A*
O3*W*—H3*WA*⋯N8^i^	0.92	1.86	2.753 (4)	162
O3*W*—H3*W*⋯N5^ii^	0.88	1.87	2.738 (4)	167
O2*W*—H2*WA*⋯O3*W*^iii^	0.85	1.88	2.723 (3)	178
O2*W*—H2*W*⋯N7^iv^	0.86	1.98	2.844 (4)	174
O1*W*—H1*W*⋯N6^v^	0.81	2.08	2.824 (4)	153
O4*W*—H4*W*⋯O4	0.85	2.22	2.975 (9)	147
O1*W*—H1*WA*⋯O3*W*	0.80	1.91	2.684 (3)	163
O4*W*—H4*WA*⋯O6	0.85	2.43	3.035 (14)	129
C23—H23⋯O4^iv^	0.93	2.53	3.379 (6)	152
C28—H28⋯O5^vi^	0.93	2.43	3.255 (11)	148
C39—H39⋯O8	0.93	2.52	3.214 (9)	132

Symmetry codes: (i) 
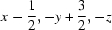
; (ii) 
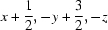
; (iii) 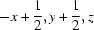; (iv) 
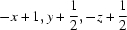
; (v) 
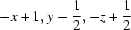
; (vi) 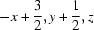.

**Fig. 1.**
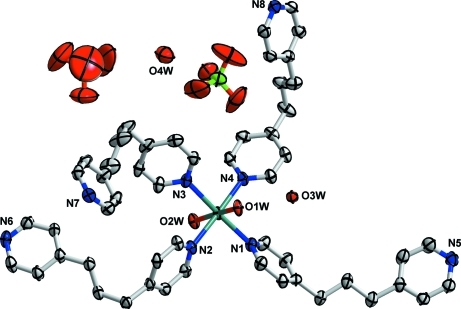
: The coordination environment of manganese ion in the title complex with the ellipsoids drawn at the 30% probability level. The hydrogen atoms are omitted for clarity.

## supplementary crystallographic information

### Comment

Flexible compounds, e.g., 1,3-di-4-pyridylpropane, are often used as ligands to synthesize complexes for their variable conformations. Herein, we report the crystal structure of thr title complex.

The asymmetric unit of the title complex consists of one manganese ion, four 1,3-di-4-pyridylpropane molecules, two coordinated water molecules, and sesqui crystallization water molecule. The Mn ion is coordinated by four N atoms from four different 1,3-di-4-pyridylpropane molecules and two O atoms from two coordinated water molecules, displaying a distorted MnN_4_O_2_ octahedral geometry (Fig. 1). Each 1,3-di-4-pyridylpropane displays a monodentate coordinating mode. In the crystal structure, there exist O—H···O, O—H···N and C—H···O hydrogen bonds (Table 1). Perchlorate anions and water molecules including coordinated water molecule and lattice water molecule, play very important roles in the formation of these hydrogen bonding interactions.

### Experimental

Reaction mixture of Mn(ClO_4_)_2_.6H_2_O (72.3 mg, 0.2 mmol), 1,3-di-4-pyridylpropane (39.6 mg, 0.2 mmol), 4-((1*H*-benzo[*d*]imidazol-1-yl)methyl)benzoic acid (50.4 mg, 0.2 mmol) and potassium hydroxide (11.2 mg, 0.2 mmol) in 12 ml H_2_O was sealed in a 16 ml Teflon-lined stainless steel container and heated to 373 K for 3 days. After cooling to room temperature, colorless block crystals of the title complex were obtained.

### Refinement

The hydrogen atoms bonded to C atoms were located in geometrically idealized positions and constrained to ride on their parent atoms, with C—H = 0.93–0.97 Å and *U*_iso_(H) = 1.2*U*_eq_(C). The hydrogen atoms of water molecules were located from difference Fourier maps and fixed at those positions with *U*_iso_(H) = 1.2*U*_eq_(O).

### Crystal data

**Table:**

[Mn(C_13_H_14_N_2_)_4_(H_2_O)_2_](ClO_4_)_2_·1.5H_2_O	*F*(000) = 4648
*M**_r_* = 1108.94	*D*_x_ = 1.291 Mg m^−^^3^
Orthorhombic, *P**b**c**a*	Mo *K*α radiation, λ = 0.71073 Å
Hall symbol: -P 2ac 2ab	Cell parameters from 8775 reflections
*a* = 19.0755 (10) Å	θ = 2.3–22.1°
*b* = 15.9409 (9) Å	µ = 0.39 mm^−^^1^
*c* = 37.533 (2) Å	*T* = 293 K
*V* = 11412.9 (11) Å^3^	Block, colorless
*Z* = 8	0.30 × 0.30 × 0.10 mm

### Data collection

**Table:**

Bruker APEXII CCD area-detector diffractometer	10065 independent reflections
Radiation source: fine-focus sealed tube	6106 reflections with *I* > 2.σ(*I*)
graphite	*R*_int_ = 0.113
ψ and ω scans	θ_max_ = 25.0°, θ_min_ = 1.5°
Absorption correction: multi-scan (SADBAS; Sheldrick, 1996)	*h* = −22→21
*T*_min_ = 0.892, *T*_max_ = 0.962	*k* = −15→18
61756 measured reflections	*l* = −44→44

### Refinement

**Table:**

Refinement on *F*^2^	Primary atom site location: structure-invariant direct methods
Least-squares matrix: full	Secondary atom site location: difference Fourier map
*R*[*F*^2^ > 2σ(*F*^2^)] = 0.057	Hydrogen site location: inferred from neighbouring sites
*wR*(*F*^2^) = 0.188	H-atom parameters constrained
*S* = 1.01	*w* = 1/[σ^2^(*F*_o_^2^) + (0.1063*P*)^2^] where *P* = (*F*_o_^2^ + 2*F*_c_^2^)/3
10065 reflections	(Δ/σ)_max_ < 0.001
676 parameters	Δρ_max_ = 0.66 e Å^−^^3^
0 restraints	Δρ_min_ = −0.47 e Å^−^^3^

### Special details

**Table:**

Geometry. All e.s.d.'s (except the e.s.d. in the dihedral angle between two l.s. planes) are estimated using the full covariance matrix. The cell e.s.d.'s are taken into account individually in the estimation of e.s.d.'s in distances, angles and torsion angles; correlations between e.s.d.'s in cell parameters are only used when they are defined by crystal symmetry. An approximate (isotropic) treatment of cell e.s.d.'s is used for estimating e.s.d.'s involving l.s. planes.
Refinement. Refinement of *F*^2^ against ALL reflections. The weighted *R*-factor *wR* and goodness of fit *S* are based on *F*^2^, conventional *R*-factors *R* are based on *F*, with *F* set to zero for negative *F*^2^. The threshold expression of *F*^2^ > σ(*F*^2^) is used only for calculating *R*-factors(gt) *etc*. and is not relevant to the choice of reflections for refinement. *R*-factors based on *F*^2^ are statistically about twice as large as those based on *F*, and *R*- factors based on ALL data will be even larger.

### Fractional atomic coordinates and isotropic or equivalent isotropic displacement parameters (Å^2^)

**Table:**

	*x*	*y*	*z*	*U*_iso_*/*U*_eq_	Occ. (<1)
C1	0.14223 (18)	1.0314 (2)	0.10488 (10)	0.0597 (10)	
H1	0.1678	1.0694	0.0913	0.072*	
C2	0.07095 (19)	1.0255 (3)	0.09937 (11)	0.0655 (11)	
H2	0.0496	1.0588	0.0822	0.079*	
C3	0.03127 (18)	0.9707 (2)	0.11906 (9)	0.0527 (9)	
C4	0.06661 (19)	0.9235 (2)	0.14426 (10)	0.0559 (9)	
H4	0.0422	0.8859	0.1586	0.067*	
C5	0.13743 (18)	0.9323 (2)	0.14805 (10)	0.0547 (9)	
H5	0.1599	0.8994	0.1651	0.066*	
C6	−0.04637 (19)	0.9611 (3)	0.11315 (11)	0.0678 (11)	
H6A	−0.0687	1.0155	0.1154	0.081*	
H6B	−0.0656	0.9247	0.1314	0.081*	
C7	−0.06281 (19)	0.9247 (2)	0.07667 (11)	0.0665 (11)	
H7A	−0.0451	0.9623	0.0584	0.080*	
H7B	−0.0389	0.8714	0.0741	0.080*	
C8	−0.1395 (2)	0.9121 (3)	0.07118 (12)	0.0882 (15)	
H8A	−0.1623	0.9664	0.0723	0.106*	
H8B	−0.1573	0.8787	0.0908	0.106*	
C9	−0.16058 (19)	0.8698 (2)	0.03651 (10)	0.0606 (10)	
C10	−0.1205 (2)	0.8723 (3)	0.00598 (12)	0.0782 (13)	
H10	−0.0778	0.9005	0.0061	0.094*	
C11	−0.1432 (2)	0.8335 (3)	−0.02461 (12)	0.0821 (13)	
H11	−0.1145	0.8350	−0.0446	0.099*	
C12	−0.2414 (2)	0.7900 (3)	0.00292 (12)	0.0712 (11)	
H12	−0.2837	0.7610	0.0023	0.085*	
C13	−0.22126 (19)	0.8261 (2)	0.03454 (11)	0.0637 (10)	
H13	−0.2494	0.8204	0.0546	0.076*	
C14	0.24921 (19)	1.0441 (2)	0.21075 (9)	0.0538 (9)	
H14	0.2154	1.0733	0.1979	0.065*	
C15	0.2552 (2)	1.0593 (2)	0.24677 (10)	0.0580 (9)	
H15	0.2262	1.0987	0.2576	0.070*	
C16	0.30397 (19)	1.0168 (2)	0.26680 (9)	0.0544 (9)	
C17	0.3431 (2)	0.9564 (2)	0.24916 (9)	0.0549 (9)	
H17	0.3747	0.9234	0.2618	0.066*	
C18	0.3351 (2)	0.9453 (2)	0.21296 (9)	0.0531 (9)	
H18	0.3628	0.9053	0.2016	0.064*	
C19	0.3159 (2)	1.0358 (3)	0.30553 (9)	0.0697 (11)	
H19A	0.3308	0.9852	0.3177	0.084*	
H19B	0.2722	1.0541	0.3162	0.084*	
C20	0.3716 (2)	1.1043 (3)	0.31056 (10)	0.0715 (12)	
H20A	0.3563	1.1545	0.2982	0.086*	
H20B	0.3749	1.1176	0.3357	0.086*	
C21	0.4431 (2)	1.0807 (3)	0.29724 (10)	0.0681 (11)	
H21A	0.4412	1.0766	0.2715	0.082*	
H21B	0.4546	1.0255	0.3064	0.082*	
C22	0.5018 (2)	1.1405 (2)	0.30718 (10)	0.0579 (9)	
C23	0.5094 (2)	1.1700 (2)	0.34110 (10)	0.0688 (11)	
H23	0.4770	1.1554	0.3585	0.083*	
C24	0.5653 (2)	1.2215 (2)	0.34953 (11)	0.0688 (11)	
H24	0.5698	1.2396	0.3730	0.083*	
C25	0.6038 (2)	1.2202 (3)	0.29304 (12)	0.0686 (11)	
H25	0.6352	1.2382	0.2757	0.082*	
C26	0.55031 (19)	1.1674 (3)	0.28279 (10)	0.0644 (10)	
H26	0.5471	1.1500	0.2592	0.077*	
C27	0.45059 (19)	1.0310 (2)	0.15797 (10)	0.0602 (10)	
H27	0.4270	1.0735	0.1699	0.072*	
C28	0.5213 (2)	1.0201 (3)	0.16459 (11)	0.0676 (11)	
H28	0.5440	1.0553	0.1806	0.081*	
C29	0.55806 (19)	0.9584 (3)	0.14793 (11)	0.0632 (11)	
C30	0.5216 (2)	0.9099 (3)	0.12349 (11)	0.0653 (11)	
H30	0.5443	0.8676	0.1110	0.078*	
C31	0.45089 (19)	0.9250 (2)	0.11788 (10)	0.0574 (9)	
H31	0.4275	0.8926	0.1010	0.069*	
C32	0.6336 (2)	0.9401 (3)	0.15606 (12)	0.0893 (15)	
H32A	0.6571	0.9916	0.1628	0.107*	
H32B	0.6564	0.9184	0.1349	0.107*	
C33	0.6403 (2)	0.8748 (4)	0.18692 (14)	0.126 (2)	
H33A	0.6192	0.8227	0.1789	0.151*	
H33B	0.6898	0.8638	0.1906	0.151*	
C34	0.6109 (3)	0.8954 (4)	0.21972 (14)	0.1048 (17)	
H34	0.5860	0.9447	0.2236	0.126*	
C35	0.6232 (2)	0.8298 (3)	0.24870 (10)	0.0683 (11)	
C36	0.5681 (2)	0.7938 (2)	0.26690 (11)	0.0674 (11)	
H36	0.5222	0.8053	0.2601	0.081*	
C37	0.5809 (2)	0.7406 (2)	0.29507 (10)	0.0597 (10)	
H37	0.5428	0.7171	0.3068	0.072*	
C38	0.6976 (2)	0.7557 (2)	0.28853 (10)	0.0579 (10)	
H38	0.7430	0.7433	0.2958	0.069*	
C39	0.68936 (19)	0.8087 (3)	0.25999 (11)	0.0680 (11)	
H39	0.7285	0.8302	0.2483	0.082*	
C40	0.3504 (2)	1.0414 (2)	0.05622 (10)	0.0584 (9)	
H40	0.3788	1.0747	0.0706	0.070*	
C41	0.3601 (2)	1.0443 (2)	0.02028 (10)	0.0649 (10)	
H41	0.3946	1.0792	0.0109	0.078*	
C42	0.3198 (2)	0.9966 (2)	−0.00230 (9)	0.0570 (10)	
C43	0.2689 (2)	0.9476 (2)	0.01370 (10)	0.0623 (10)	
H43	0.2391	0.9152	−0.0003	0.075*	
C44	0.2621 (2)	0.9468 (2)	0.05040 (9)	0.0560 (9)	
H44	0.2282	0.9123	0.0605	0.067*	
C45	0.3314 (3)	0.9972 (3)	−0.04187 (9)	0.0790 (13)	
H45A	0.3347	1.0548	−0.0500	0.095*	
H45B	0.2914	0.9716	−0.0535	0.095*	
C46	0.3973 (2)	0.9506 (2)	−0.05256 (11)	0.0757 (12)	
H46A	0.4057	0.9598	−0.0777	0.091*	
H46B	0.4367	0.9740	−0.0396	0.091*	
C47	0.3943 (2)	0.8574 (2)	−0.04568 (11)	0.0706 (11)	
H47A	0.3559	0.8334	−0.0592	0.085*	
H47B	0.3850	0.8478	−0.0206	0.085*	
C48	0.46184 (19)	0.8138 (2)	−0.05589 (10)	0.0554 (9)	
C49	0.5060 (2)	0.7804 (2)	−0.03079 (10)	0.0636 (10)	
H49	0.4938	0.7823	−0.0068	0.076*	
C50	0.5681 (2)	0.7442 (3)	−0.04085 (11)	0.0697 (11)	
H50	0.5966	0.7210	−0.0233	0.084*	
C51	0.5469 (2)	0.7728 (2)	−0.09890 (11)	0.0647 (10)	
H51	0.5606	0.7709	−0.1227	0.078*	
C52	0.4838 (2)	0.8086 (2)	−0.09083 (10)	0.0660 (10)	
H52	0.4555	0.8295	−0.1090	0.079*	
Cl1	0.58949 (8)	0.70331 (11)	0.06219 (3)	0.1041 (5)	
Cl2	0.86174 (6)	0.74954 (9)	0.18277 (3)	0.0814 (4)	
Mn1	0.29477 (3)	0.98755 (3)	0.132673 (12)	0.04169 (17)	
N1	0.17626 (15)	0.98481 (17)	0.12894 (7)	0.0478 (7)	
N2	0.28962 (14)	0.98929 (16)	0.19338 (7)	0.0477 (7)	
N3	0.41480 (15)	0.98328 (17)	0.13525 (7)	0.0492 (7)	
N4	0.30208 (15)	0.99315 (16)	0.07197 (7)	0.0487 (7)	
N5	−0.20398 (19)	0.7941 (2)	−0.02681 (9)	0.0730 (9)	
N6	0.61299 (18)	1.24651 (18)	0.32579 (10)	0.0642 (9)	
N7	0.64522 (17)	0.72116 (18)	0.30637 (8)	0.0565 (8)	
N8	0.58976 (18)	0.7405 (2)	−0.07466 (9)	0.0672 (9)	
O1	0.6203 (5)	0.6491 (4)	0.03846 (15)	0.266 (4)	
O2	0.5196 (3)	0.6942 (5)	0.06484 (15)	0.225 (3)	
O3	0.6027 (2)	0.7825 (3)	0.04889 (12)	0.1479 (16)	
O4	0.6163 (3)	0.6917 (5)	0.09613 (11)	0.230 (3)	
O5	0.8830 (6)	0.6780 (5)	0.1902 (4)	0.386 (7)	
O6	0.8360 (5)	0.7531 (9)	0.1523 (2)	0.402 (8)	
O7	0.9184 (3)	0.7981 (6)	0.1854 (2)	0.285 (4)	
O8	0.8137 (4)	0.7648 (5)	0.2053 (2)	0.283 (4)	
O1W	0.29594 (11)	0.85152 (13)	0.13508 (6)	0.0529 (6)	
H1W	0.3101	0.8191	0.1501	0.063*	
H1WA	0.2837	0.8222	0.1189	0.063*	
O2W	0.29751 (12)	1.12426 (13)	0.13705 (5)	0.0518 (6)	
H2W	0.3165	1.1558	0.1531	0.062*	
H2WA	0.2894	1.1621	0.1218	0.062*	
O3W	0.23088 (12)	0.74826 (13)	0.08930 (6)	0.0526 (6)	
H3W	0.2567	0.7409	0.0700	0.063*	
H3WA	0.1848	0.7636	0.0858	0.063*	
O4W	0.7562 (4)	0.6184 (6)	0.1121 (2)	0.130 (3)	0.50
H4WA	0.7719	0.6680	0.1096	0.156*	0.50
H4W	0.7137	0.6182	0.1055	0.156*	0.50

### Atomic displacement parameters (Å^2^)

**Table:**

	*U*^11^	*U*^22^	*U*^33^	*U*^12^	*U*^13^	*U*^23^
C1	0.048 (2)	0.056 (2)	0.075 (3)	0.0006 (17)	−0.0087 (19)	0.016 (2)
C2	0.052 (2)	0.072 (3)	0.072 (3)	0.006 (2)	−0.013 (2)	0.020 (2)
C3	0.049 (2)	0.055 (2)	0.055 (2)	−0.0003 (18)	−0.0047 (18)	−0.0070 (18)
C4	0.059 (2)	0.051 (2)	0.058 (2)	−0.0056 (18)	−0.0051 (19)	0.0011 (18)
C5	0.052 (2)	0.052 (2)	0.060 (2)	0.0007 (17)	−0.0087 (18)	0.0110 (18)
C6	0.049 (2)	0.085 (3)	0.070 (2)	−0.006 (2)	−0.012 (2)	−0.007 (2)
C7	0.052 (2)	0.061 (2)	0.086 (3)	−0.0010 (19)	−0.018 (2)	−0.007 (2)
C8	0.058 (3)	0.116 (4)	0.090 (3)	−0.004 (2)	−0.016 (2)	−0.025 (3)
C9	0.054 (2)	0.068 (3)	0.060 (2)	0.005 (2)	−0.0147 (19)	0.004 (2)
C10	0.067 (3)	0.091 (3)	0.077 (3)	−0.021 (2)	−0.017 (2)	0.015 (3)
C11	0.072 (3)	0.111 (4)	0.063 (3)	−0.004 (3)	−0.011 (2)	0.024 (3)
C12	0.060 (2)	0.077 (3)	0.077 (3)	−0.004 (2)	−0.008 (2)	−0.007 (2)
C13	0.055 (2)	0.066 (2)	0.070 (3)	0.001 (2)	−0.004 (2)	−0.007 (2)
C14	0.057 (2)	0.054 (2)	0.050 (2)	−0.0011 (18)	−0.0057 (18)	0.0000 (18)
C15	0.059 (2)	0.058 (2)	0.056 (2)	−0.0079 (19)	0.0057 (19)	−0.0087 (19)
C16	0.059 (2)	0.061 (2)	0.0433 (19)	−0.0275 (19)	−0.0027 (17)	0.0038 (18)
C17	0.064 (2)	0.049 (2)	0.051 (2)	−0.0090 (18)	−0.0168 (19)	0.0069 (18)
C18	0.069 (2)	0.042 (2)	0.048 (2)	−0.0008 (17)	−0.0135 (19)	−0.0002 (16)
C19	0.069 (3)	0.096 (3)	0.044 (2)	−0.030 (2)	0.0012 (19)	−0.005 (2)
C20	0.073 (3)	0.087 (3)	0.055 (2)	−0.020 (2)	−0.007 (2)	−0.020 (2)
C21	0.073 (3)	0.073 (3)	0.059 (2)	−0.025 (2)	−0.001 (2)	−0.014 (2)
C22	0.064 (2)	0.054 (2)	0.056 (2)	−0.0164 (19)	−0.0099 (19)	−0.0044 (18)
C23	0.085 (3)	0.065 (3)	0.057 (2)	−0.028 (2)	−0.002 (2)	−0.007 (2)
C24	0.092 (3)	0.057 (2)	0.058 (2)	−0.018 (2)	−0.017 (2)	−0.007 (2)
C25	0.061 (2)	0.072 (3)	0.072 (3)	−0.014 (2)	−0.001 (2)	0.001 (2)
C26	0.059 (2)	0.077 (3)	0.057 (2)	−0.017 (2)	−0.0062 (19)	−0.012 (2)
C27	0.059 (2)	0.046 (2)	0.076 (3)	−0.0040 (18)	−0.019 (2)	0.0026 (19)
C28	0.066 (3)	0.055 (2)	0.081 (3)	−0.019 (2)	−0.024 (2)	0.021 (2)
C29	0.048 (2)	0.074 (3)	0.068 (2)	−0.007 (2)	−0.005 (2)	0.040 (2)
C30	0.059 (2)	0.070 (3)	0.066 (2)	0.015 (2)	0.009 (2)	0.019 (2)
C31	0.056 (2)	0.058 (2)	0.059 (2)	0.0009 (19)	−0.0041 (19)	0.0006 (19)
C32	0.050 (2)	0.125 (4)	0.093 (3)	−0.004 (2)	−0.002 (2)	0.060 (3)
C33	0.061 (3)	0.216 (7)	0.100 (4)	0.051 (4)	0.002 (3)	0.071 (4)
C34	0.095 (4)	0.125 (4)	0.095 (4)	0.037 (3)	−0.002 (3)	0.040 (3)
C35	0.057 (2)	0.080 (3)	0.068 (2)	0.000 (2)	−0.007 (2)	0.029 (2)
C36	0.054 (2)	0.071 (3)	0.077 (3)	−0.002 (2)	−0.015 (2)	0.016 (2)
C37	0.058 (2)	0.061 (2)	0.061 (2)	0.0013 (19)	−0.0011 (19)	0.0071 (19)
C38	0.052 (2)	0.058 (2)	0.064 (2)	0.0068 (18)	−0.0157 (19)	0.0024 (19)
C39	0.051 (2)	0.082 (3)	0.072 (3)	−0.001 (2)	−0.004 (2)	0.023 (2)
C40	0.069 (2)	0.047 (2)	0.060 (2)	−0.0074 (19)	−0.003 (2)	−0.0025 (18)
C41	0.078 (3)	0.054 (2)	0.063 (2)	−0.005 (2)	0.013 (2)	0.005 (2)
C42	0.080 (3)	0.046 (2)	0.045 (2)	0.018 (2)	−0.0022 (19)	0.0020 (17)
C43	0.082 (3)	0.053 (2)	0.052 (2)	−0.003 (2)	−0.016 (2)	−0.0100 (19)
C44	0.070 (2)	0.048 (2)	0.050 (2)	−0.0076 (18)	−0.0087 (19)	−0.0004 (17)
C45	0.125 (4)	0.063 (3)	0.049 (2)	0.031 (3)	0.005 (2)	0.0085 (19)
C46	0.109 (3)	0.059 (2)	0.060 (2)	−0.001 (2)	0.024 (2)	0.010 (2)
C47	0.077 (3)	0.056 (2)	0.079 (3)	0.002 (2)	0.017 (2)	0.011 (2)
C48	0.065 (2)	0.044 (2)	0.057 (2)	−0.0059 (18)	0.0084 (19)	0.0027 (17)
C49	0.074 (3)	0.071 (3)	0.046 (2)	−0.009 (2)	0.011 (2)	−0.0031 (19)
C50	0.066 (3)	0.086 (3)	0.058 (2)	−0.006 (2)	−0.003 (2)	0.000 (2)
C51	0.077 (3)	0.066 (3)	0.051 (2)	−0.006 (2)	0.009 (2)	−0.007 (2)
C52	0.077 (3)	0.063 (3)	0.058 (2)	−0.006 (2)	0.002 (2)	0.002 (2)
Cl1	0.1142 (11)	0.1330 (12)	0.0652 (8)	−0.0252 (9)	0.0104 (7)	0.0228 (8)
Cl2	0.0720 (8)	0.1004 (9)	0.0717 (7)	0.0177 (7)	0.0082 (6)	−0.0079 (6)
Mn1	0.0469 (3)	0.0362 (3)	0.0419 (3)	0.0010 (2)	−0.0107 (2)	−0.0012 (2)
N1	0.0469 (16)	0.0448 (16)	0.0518 (17)	0.0034 (14)	−0.0098 (14)	0.0053 (14)
N2	0.0593 (18)	0.0384 (15)	0.0456 (15)	−0.0017 (14)	−0.0083 (14)	−0.0028 (13)
N3	0.0478 (16)	0.0412 (16)	0.0587 (18)	0.0004 (14)	−0.0116 (14)	0.0026 (14)
N4	0.0613 (18)	0.0399 (16)	0.0451 (16)	−0.0013 (14)	−0.0070 (14)	0.0004 (13)
N5	0.070 (2)	0.081 (2)	0.069 (2)	0.0043 (19)	−0.0157 (19)	−0.0024 (19)
N6	0.067 (2)	0.0501 (19)	0.075 (2)	−0.0130 (16)	−0.0197 (19)	0.0016 (17)
N7	0.064 (2)	0.0494 (18)	0.0561 (18)	0.0057 (15)	−0.0063 (16)	0.0028 (15)
N8	0.067 (2)	0.069 (2)	0.066 (2)	−0.0103 (17)	0.0092 (18)	−0.0078 (18)
O1	0.499 (13)	0.146 (5)	0.152 (5)	0.097 (6)	0.104 (6)	0.003 (4)
O2	0.122 (4)	0.342 (9)	0.211 (6)	−0.116 (5)	−0.030 (4)	0.133 (6)
O3	0.157 (4)	0.136 (4)	0.150 (4)	−0.051 (3)	0.030 (3)	0.022 (3)
O4	0.161 (5)	0.443 (11)	0.086 (3)	0.041 (5)	−0.005 (3)	0.073 (4)
O5	0.420 (15)	0.138 (6)	0.60 (2)	0.134 (8)	−0.031 (12)	−0.032 (9)
O6	0.262 (9)	0.78 (2)	0.165 (6)	−0.151 (11)	−0.110 (7)	0.125 (9)
O7	0.124 (4)	0.351 (11)	0.379 (11)	−0.081 (6)	−0.027 (5)	0.110 (9)
O8	0.205 (6)	0.350 (11)	0.293 (9)	0.006 (6)	0.136 (6)	−0.104 (8)
O1W	0.0661 (15)	0.0344 (12)	0.0582 (14)	−0.0003 (11)	−0.0221 (12)	−0.0011 (10)
O2W	0.0750 (16)	0.0336 (12)	0.0469 (13)	−0.0014 (11)	−0.0150 (11)	0.0014 (10)
O3W	0.0577 (14)	0.0523 (14)	0.0479 (13)	−0.0098 (11)	−0.0016 (11)	−0.0021 (11)
O4W	0.132 (6)	0.141 (7)	0.118 (6)	0.020 (5)	0.017 (5)	−0.014 (5)

### Geometric parameters (Å, °)

**Table:**

C1—N1	1.337 (4)	C32—C33	1.563 (6)
C1—C2	1.379 (5)	C32—H32A	0.9700
C1—H1	0.9300	C32—H32B	0.9700
C2—C3	1.372 (5)	C33—C34	1.392 (7)
C2—H2	0.9300	C33—H33A	0.9700
C3—C4	1.383 (5)	C33—H33B	0.9700
C3—C6	1.505 (5)	C34—C35	1.527 (6)
C4—C5	1.366 (5)	C34—H34	0.9300
C4—H4	0.9300	C35—C39	1.372 (5)
C5—N1	1.328 (4)	C35—C36	1.379 (5)
C5—H5	0.9300	C36—C37	1.376 (5)
C6—C7	1.520 (5)	C36—H36	0.9300
C6—H6A	0.9700	C37—N7	1.334 (5)
C6—H6B	0.9700	C37—H37	0.9300
C7—C8	1.491 (5)	C38—N7	1.323 (5)
C7—H7A	0.9700	C38—C39	1.373 (5)
C7—H7B	0.9700	C38—H38	0.9300
C8—C9	1.520 (5)	C39—H39	0.9300
C8—H8A	0.9700	C40—N4	1.338 (4)
C8—H8B	0.9700	C40—C41	1.363 (5)
C9—C13	1.353 (5)	C40—H40	0.9300
C9—C10	1.378 (5)	C41—C42	1.374 (5)
C10—C11	1.374 (6)	C41—H41	0.9300
C10—H10	0.9300	C42—C43	1.384 (5)
C11—N5	1.322 (5)	C42—C45	1.502 (5)
C11—H11	0.9300	C43—C44	1.383 (5)
C12—N5	1.326 (5)	C43—H43	0.9300
C12—C13	1.374 (5)	C44—N4	1.335 (4)
C12—H12	0.9300	C44—H44	0.9300
C13—H13	0.9300	C45—C46	1.513 (6)
C14—N2	1.335 (4)	C45—H45A	0.9700
C14—C15	1.379 (5)	C45—H45B	0.9700
C14—H14	0.9300	C46—C47	1.509 (5)
C15—C16	1.375 (5)	C46—H46A	0.9700
C15—H15	0.9300	C46—H46B	0.9700
C16—C17	1.386 (5)	C47—C48	1.514 (5)
C16—C19	1.502 (5)	C47—H47A	0.9700
C17—C18	1.378 (4)	C47—H47B	0.9700
C17—H17	0.9300	C48—C49	1.371 (5)
C18—N2	1.336 (4)	C48—C52	1.379 (5)
C18—H18	0.9300	C49—C50	1.371 (5)
C19—C20	1.534 (5)	C49—H49	0.9300
C19—H19A	0.9700	C50—N8	1.335 (5)
C19—H19B	0.9700	C50—H50	0.9300
C20—C21	1.500 (6)	C51—N8	1.327 (5)
C20—H20A	0.9700	C51—C52	1.367 (5)
C20—H20B	0.9700	C51—H51	0.9300
C21—C22	1.518 (5)	C52—H52	0.9300
C21—H21A	0.9700	Cl1—O2	1.344 (5)
C21—H21B	0.9700	Cl1—O1	1.373 (5)
C22—C23	1.365 (5)	Cl1—O3	1.381 (4)
C22—C26	1.371 (5)	Cl1—O4	1.385 (4)
C23—C24	1.381 (5)	Cl2—O5	1.242 (7)
C23—H23	0.9300	Cl2—O6	1.245 (6)
C24—N6	1.335 (5)	Cl2—O8	1.271 (6)
C24—H24	0.9300	Cl2—O7	1.334 (6)
C25—N6	1.311 (5)	Mn1—O1W	2.170 (2)
C25—C26	1.376 (5)	Mn1—O2W	2.186 (2)
C25—H25	0.9300	Mn1—N1	2.265 (3)
C26—H26	0.9300	Mn1—N2	2.281 (3)
C27—N3	1.331 (4)	Mn1—N4	2.284 (3)
C27—C28	1.383 (5)	Mn1—N3	2.293 (3)
C27—H27	0.9300	O1W—H1W	0.8104
C28—C29	1.359 (6)	O1W—H1WA	0.8011
C28—H28	0.9300	O2W—H2W	0.8636
C29—C30	1.387 (6)	O2W—H2WA	0.8457
C29—C32	1.502 (5)	O3W—H3W	0.8847
C30—C31	1.386 (5)	O3W—H3WA	0.9219
C30—H30	0.9300	O4W—H4WA	0.8497
C31—N3	1.327 (4)	O4W—H4W	0.8491
C31—H31	0.9300		
			
N1—C1—C2	122.8 (3)	C34—C33—H33B	107.9
N1—C1—H1	118.6	C32—C33—H33B	107.9
C2—C1—H1	118.6	H33A—C33—H33B	107.2
C3—C2—C1	120.4 (3)	C33—C34—C35	114.0 (5)
C3—C2—H2	119.8	C33—C34—H34	123.0
C1—C2—H2	119.8	C35—C34—H34	123.0
C2—C3—C4	116.5 (3)	C39—C35—C36	116.5 (3)
C2—C3—C6	121.8 (3)	C39—C35—C34	121.9 (4)
C4—C3—C6	121.7 (3)	C36—C35—C34	121.4 (4)
C5—C4—C3	119.8 (3)	C37—C36—C35	120.1 (4)
C5—C4—H4	120.1	C37—C36—H36	120.0
C3—C4—H4	120.1	C35—C36—H36	120.0
N1—C5—C4	124.0 (3)	N7—C37—C36	123.4 (4)
N1—C5—H5	118.0	N7—C37—H37	118.3
C4—C5—H5	118.0	C36—C37—H37	118.3
C3—C6—C7	112.0 (3)	N7—C38—C39	124.4 (3)
C3—C6—H6A	109.2	N7—C38—H38	117.8
C7—C6—H6A	109.2	C39—C38—H38	117.8
C3—C6—H6B	109.2	C35—C39—C38	119.8 (4)
C7—C6—H6B	109.2	C35—C39—H39	120.1
H6A—C6—H6B	107.9	C38—C39—H39	120.1
C8—C7—C6	112.2 (3)	N4—C40—C41	123.5 (4)
C8—C7—H7A	109.2	N4—C40—H40	118.3
C6—C7—H7A	109.2	C41—C40—H40	118.3
C8—C7—H7B	109.2	C40—C41—C42	121.0 (4)
C6—C7—H7B	109.2	C40—C41—H41	119.5
H7A—C7—H7B	107.9	C42—C41—H41	119.5
C7—C8—C9	116.0 (4)	C41—C42—C43	116.0 (3)
C7—C8—H8A	108.3	C41—C42—C45	121.6 (4)
C9—C8—H8A	108.3	C43—C42—C45	122.4 (4)
C7—C8—H8B	108.3	C44—C43—C42	120.2 (3)
C9—C8—H8B	108.3	C44—C43—H43	119.9
H8A—C8—H8B	107.4	C42—C43—H43	119.9
C13—C9—C10	116.4 (4)	N4—C44—C43	123.1 (3)
C13—C9—C8	120.1 (4)	N4—C44—H44	118.5
C10—C9—C8	123.5 (4)	C43—C44—H44	118.5
C11—C10—C9	120.5 (4)	C42—C45—C46	112.5 (3)
C11—C10—H10	119.8	C42—C45—H45A	109.1
C9—C10—H10	119.8	C46—C45—H45A	109.1
N5—C11—C10	122.9 (4)	C42—C45—H45B	109.1
N5—C11—H11	118.6	C46—C45—H45B	109.1
C10—C11—H11	118.6	H45A—C45—H45B	107.8
N5—C12—C13	123.8 (4)	C47—C46—C45	113.9 (3)
N5—C12—H12	118.1	C47—C46—H46A	108.8
C13—C12—H12	118.1	C45—C46—H46A	108.8
C9—C13—C12	120.1 (4)	C47—C46—H46B	108.8
C9—C13—H13	119.9	C45—C46—H46B	108.8
C12—C13—H13	119.9	H46A—C46—H46B	107.7
N2—C14—C15	123.1 (3)	C46—C47—C48	112.2 (3)
N2—C14—H14	118.4	C46—C47—H47A	109.2
C15—C14—H14	118.4	C48—C47—H47A	109.2
C16—C15—C14	120.3 (4)	C46—C47—H47B	109.2
C16—C15—H15	119.8	C48—C47—H47B	109.2
C14—C15—H15	119.8	H47A—C47—H47B	107.9
C15—C16—C17	116.5 (3)	C49—C48—C52	116.3 (4)
C15—C16—C19	122.1 (4)	C49—C48—C47	121.8 (3)
C17—C16—C19	121.4 (4)	C52—C48—C47	121.8 (4)
C18—C17—C16	120.1 (3)	C50—C49—C48	120.4 (4)
C18—C17—H17	120.0	C50—C49—H49	119.8
C16—C17—H17	120.0	C48—C49—H49	119.8
N2—C18—C17	123.1 (3)	N8—C50—C49	123.2 (4)
N2—C18—H18	118.5	N8—C50—H50	118.4
C17—C18—H18	118.5	C49—C50—H50	118.4
C16—C19—C20	111.6 (3)	N8—C51—C52	123.5 (4)
C16—C19—H19A	109.3	N8—C51—H51	118.2
C20—C19—H19A	109.3	C52—C51—H51	118.2
C16—C19—H19B	109.3	C51—C52—C48	120.2 (4)
C20—C19—H19B	109.3	C51—C52—H52	119.9
H19A—C19—H19B	108.0	C48—C52—H52	119.9
C21—C20—C19	114.2 (4)	O2—Cl1—O1	113.8 (5)
C21—C20—H20A	108.7	O2—Cl1—O3	107.9 (4)
C19—C20—H20A	108.7	O1—Cl1—O3	105.3 (3)
C21—C20—H20B	108.7	O2—Cl1—O4	106.5 (3)
C19—C20—H20B	108.7	O1—Cl1—O4	110.8 (4)
H20A—C20—H20B	107.6	O3—Cl1—O4	112.7 (4)
C20—C21—C22	115.6 (3)	O5—Cl2—O6	112.1 (8)
C20—C21—H21A	108.4	O5—Cl2—O8	105.2 (7)
C22—C21—H21A	108.4	O6—Cl2—O8	108.5 (7)
C20—C21—H21B	108.4	O5—Cl2—O7	104.6 (6)
C22—C21—H21B	108.4	O6—Cl2—O7	111.2 (6)
H21A—C21—H21B	107.4	O8—Cl2—O7	115.0 (6)
C23—C22—C26	116.3 (3)	O1W—Mn1—O2W	173.02 (8)
C23—C22—C21	121.6 (3)	O1W—Mn1—N1	89.63 (9)
C26—C22—C21	122.1 (3)	O2W—Mn1—N1	92.74 (9)
C22—C23—C24	120.0 (4)	O1W—Mn1—N2	88.34 (9)
C22—C23—H23	120.0	O2W—Mn1—N2	85.05 (9)
C24—C23—H23	120.0	N1—Mn1—N2	91.08 (10)
N6—C24—C23	123.4 (4)	O1W—Mn1—N4	94.59 (9)
N6—C24—H24	118.3	O2W—Mn1—N4	91.98 (9)
C23—C24—H24	118.3	N1—Mn1—N4	90.00 (10)
N6—C25—C26	123.9 (4)	N2—Mn1—N4	176.89 (10)
N6—C25—H25	118.1	O1W—Mn1—N3	87.61 (9)
C26—C25—H25	118.1	O2W—Mn1—N3	90.15 (9)
C22—C26—C25	120.3 (4)	N1—Mn1—N3	176.97 (10)
C22—C26—H26	119.8	N2—Mn1—N3	90.08 (10)
C25—C26—H26	119.8	N4—Mn1—N3	88.98 (10)
N3—C27—C28	122.9 (4)	C5—N1—C1	116.4 (3)
N3—C27—H27	118.5	C5—N1—Mn1	122.4 (2)
C28—C27—H27	118.5	C1—N1—Mn1	121.0 (2)
C29—C28—C27	120.7 (4)	C14—N2—C18	116.7 (3)
C29—C28—H28	119.6	C14—N2—Mn1	121.4 (2)
C27—C28—H28	119.6	C18—N2—Mn1	121.0 (2)
C28—C29—C30	116.7 (4)	C31—N3—C27	116.6 (3)
C28—C29—C32	122.8 (4)	C31—N3—Mn1	121.2 (2)
C30—C29—C32	120.5 (4)	C27—N3—Mn1	121.5 (2)
C31—C30—C29	119.4 (4)	C44—N4—C40	116.3 (3)
C31—C30—H30	120.3	C44—N4—Mn1	123.3 (2)
C29—C30—H30	120.3	C40—N4—Mn1	120.4 (2)
N3—C31—C30	123.5 (4)	C11—N5—C12	116.3 (4)
N3—C31—H31	118.2	C25—N6—C24	116.0 (3)
C30—C31—H31	118.2	C38—N7—C37	115.9 (3)
C29—C32—C33	111.0 (3)	C51—N8—C50	116.4 (4)
C29—C32—H32A	109.4	Mn1—O1W—H1W	132.0
C33—C32—H32A	109.4	Mn1—O1W—H1WA	123.2
C29—C32—H32B	109.4	H1W—O1W—H1WA	104.7
C33—C32—H32B	109.4	Mn1—O2W—H2W	130.0
H32A—C32—H32B	108.0	Mn1—O2W—H2WA	130.9
C34—C33—C32	117.7 (5)	H2W—O2W—H2WA	97.7
C34—C33—H33A	107.9	H3W—O3W—H3WA	116.6
C32—C33—H33A	107.9	H4WA—O4W—H4W	108.0
			
N1—C1—C2—C3	0.5 (6)	C46—C47—C48—C49	110.6 (4)
C1—C2—C3—C4	0.4 (6)	C46—C47—C48—C52	−66.5 (5)
C1—C2—C3—C6	−178.5 (4)	C52—C48—C49—C50	−0.1 (5)
C2—C3—C4—C5	−0.9 (5)	C47—C48—C49—C50	−177.4 (4)
C6—C3—C4—C5	178.0 (3)	C48—C49—C50—N8	1.3 (6)
C3—C4—C5—N1	0.6 (6)	N8—C51—C52—C48	1.0 (6)
C2—C3—C6—C7	65.5 (5)	C49—C48—C52—C51	−1.0 (5)
C4—C3—C6—C7	−113.3 (4)	C47—C48—C52—C51	176.3 (4)
C3—C6—C7—C8	177.8 (4)	C4—C5—N1—C1	0.3 (5)
C6—C7—C8—C9	−175.6 (4)	C4—C5—N1—Mn1	−173.9 (3)
C7—C8—C9—C13	153.6 (4)	C2—C1—N1—C5	−0.9 (5)
C7—C8—C9—C10	−25.7 (6)	C2—C1—N1—Mn1	173.5 (3)
C13—C9—C10—C11	1.4 (6)	O1W—Mn1—N1—C5	40.4 (3)
C8—C9—C10—C11	−179.2 (4)	O2W—Mn1—N1—C5	−133.0 (3)
C9—C10—C11—N5	1.7 (7)	N2—Mn1—N1—C5	−47.9 (3)
C10—C9—C13—C12	−2.5 (6)	N3—Mn1—N1—C5	64.6 (19)
C8—C9—C13—C12	178.0 (4)	O1W—Mn1—N1—C1	−133.6 (3)
N5—C12—C13—C9	0.9 (7)	O2W—Mn1—N1—C1	53.0 (3)
N2—C14—C15—C16	−0.8 (5)	N2—Mn1—N1—C1	138.1 (3)
C14—C15—C16—C17	−2.9 (5)	N4—Mn1—N1—C1	−39.0 (3)
C14—C15—C16—C19	175.5 (3)	C15—C14—N2—C18	3.3 (5)
C15—C16—C17—C18	4.0 (5)	C15—C14—N2—Mn1	−166.0 (3)
C19—C16—C17—C18	−174.4 (3)	C17—C18—N2—C14	−2.2 (5)
C16—C17—C18—N2	−1.5 (5)	C17—C18—N2—Mn1	167.2 (3)
C15—C16—C19—C20	−89.2 (4)	O1W—Mn1—N2—C14	−140.1 (3)
C17—C16—C19—C20	89.1 (5)	O2W—Mn1—N2—C14	42.2 (2)
C16—C19—C20—C21	−62.9 (5)	N1—Mn1—N2—C14	−50.5 (3)
C19—C20—C21—C22	−170.3 (3)	N3—Mn1—N2—C14	132.3 (3)
C20—C21—C22—C23	46.7 (6)	O1W—Mn1—N2—C18	51.0 (2)
C20—C21—C22—C26	−133.8 (4)	O2W—Mn1—N2—C18	−126.7 (2)
C26—C22—C23—C24	−2.3 (6)	N1—Mn1—N2—C18	140.6 (2)
C21—C22—C23—C24	177.2 (4)	N3—Mn1—N2—C18	−36.6 (2)
C22—C23—C24—N6	1.5 (6)	C30—C31—N3—C27	2.9 (5)
C23—C22—C26—C25	1.0 (6)	C30—C31—N3—Mn1	−167.8 (3)
C21—C22—C26—C25	−178.5 (4)	C28—C27—N3—C31	−2.1 (5)
N6—C25—C26—C22	1.3 (6)	C28—C27—N3—Mn1	168.6 (3)
N3—C27—C28—C29	−0.4 (6)	O1W—Mn1—N3—C31	39.2 (3)
C27—C28—C29—C30	2.1 (5)	O2W—Mn1—N3—C31	−147.4 (3)
C27—C28—C29—C32	−175.6 (3)	N2—Mn1—N3—C31	127.5 (3)
C28—C29—C30—C31	−1.3 (5)	N4—Mn1—N3—C31	−55.4 (3)
C32—C29—C30—C31	176.5 (3)	O1W—Mn1—N3—C27	−131.1 (3)
C29—C30—C31—N3	−1.3 (6)	O2W—Mn1—N3—C27	42.3 (3)
C28—C29—C32—C33	89.2 (6)	N2—Mn1—N3—C27	−42.8 (3)
C30—C29—C32—C33	−88.4 (5)	N4—Mn1—N3—C27	134.2 (3)
C29—C32—C33—C34	−59.5 (7)	C43—C44—N4—C40	−0.3 (5)
C32—C33—C34—C35	−177.1 (4)	C43—C44—N4—Mn1	−177.0 (3)
C33—C34—C35—C39	61.5 (7)	C41—C40—N4—C44	−0.4 (5)
C33—C34—C35—C36	−123.9 (5)	C41—C40—N4—Mn1	176.4 (3)
C39—C35—C36—C37	0.9 (6)	O1W—Mn1—N4—C44	47.2 (3)
C34—C35—C36—C37	−174.0 (4)	O2W—Mn1—N4—C44	−135.1 (3)
C35—C36—C37—N7	0.1 (6)	N1—Mn1—N4—C44	−42.4 (3)
C36—C35—C39—C38	−1.4 (6)	N3—Mn1—N4—C44	134.8 (3)
C34—C35—C39—C38	173.5 (4)	O1W—Mn1—N4—C40	−129.3 (3)
N7—C38—C39—C35	1.1 (7)	O2W—Mn1—N4—C40	48.3 (3)
N4—C40—C41—C42	0.0 (6)	N2—Mn1—N4—C40	31 (2)
C40—C41—C42—C43	1.1 (5)	N3—Mn1—N4—C40	−41.8 (3)
C40—C41—C42—C45	−178.1 (4)	C10—C11—N5—C12	−3.3 (7)
C41—C42—C43—C44	−1.8 (5)	C13—C12—N5—C11	2.1 (6)
C45—C42—C43—C44	177.4 (3)	C26—C25—N6—C24	−2.2 (6)
C42—C43—C44—N4	1.5 (6)	C23—C24—N6—C25	0.8 (6)
C41—C42—C45—C46	73.0 (5)	C39—C38—N7—C37	0.0 (6)
C43—C42—C45—C46	−106.1 (5)	C36—C37—N7—C38	−0.6 (6)
C42—C45—C46—C47	66.6 (5)	C52—C51—N8—C50	0.2 (6)
C45—C46—C47—C48	−178.6 (4)	C49—C50—N8—C51	−1.3 (6)

### Hydrogen-bond geometry (Å, °)

**Table:**

*D*—H···*A*	*D*—H	H···*A*	*D*···*A*	*D*—H···*A*
O3W—H3WA···N8^i^	0.92	1.86	2.753 (4)	162.
O3W—H3W···N5^ii^	0.88	1.87	2.738 (4)	167.
O2W—H2WA···O3W^iii^	0.85	1.88	2.723 (3)	178.
O2W—H2W···N7^iv^	0.86	1.98	2.844 (4)	174.
O1W—H1W···N6^v^	0.81	2.08	2.824 (4)	153.
O4W—H4W···O4	0.85	2.22	2.975 (9)	147.
O1W—H1WA···O3W	0.80	1.91	2.684 (3)	163.
O4W—H4WA···O6	0.85	2.43	3.035 (14)	129.
C23—H23···O4^iv^	0.93	2.53	3.379 (6)	152.
C28—H28···O5^vi^	0.93	2.43	3.255 (11)	148.
C39—H39···O8	0.93	2.52	3.214 (9)	132.

Symmetry codes: (i) *x*−1/2, −*y*+3/2, −*z*; (ii) *x*+1/2, −*y*+3/2, −*z*; (iii) −*x*+1/2, *y*+1/2, *z*; (iv) −*x*+1, *y*+1/2, −*z*+1/2; (v) −*x*+1, *y*−1/2, −*z*+1/2; (vi) −*x*+3/2, *y*+1/2, *z*.
